# The effect of an active on-ward participation of hospital pharmacists in Internal Medicine teams on preventable Adverse Drug Events in elderly inpatients: protocol of the WINGS study (Ward-oriented pharmacy in newly admitted geriatric seniors)

**DOI:** 10.1186/1472-6963-11-124

**Published:** 2011-05-25

**Authors:** Joanna E Klopotowska, Peter C Wierenga, Sophia E de Rooij, Clementine C Stuijt, Lambertus Arisz, Paul F Kuks, Marcel G Dijkgraaf, Loraine Lie-A-Huen, Susanne M Smorenburg

**Affiliations:** 1 Department of Hospital Pharmacy, Academic Medical Centre, Amsterdam, The Netherlands; 2 Department of Internal Medicine, Division of Geriatrics, Academic Medical Centre, Amsterdam, The Netherlands; 3 Department of Internal Medicine, Academic Medical Centre, Amsterdam, The Netherlands; 4 Clinical Research Unit, Academic Medical Centre, Amsterdam, The Netherlands; 5 Department of Quality and Process Innovation, Academic Medical Centre, Amsterdam, The Netherlands

## Abstract

**Background:**

The potential of clinical interventions, aiming at reduction of preventable Adverse Drug Events (preventable ADEs) during hospital stay, have been studied extensively. Clinical Pharmacy is a well-established and effective service, usually consisting of full-time on-ward participation of clinical pharmacists in medical teams. Within the current Hospital Pharmacy organisation in the Netherlands, such on-ward service is less feasible and therefore not yet established. However, given the substantial incidence of preventable ADEs in Dutch hospitals found in recent studies, appears warranted. Therefore, "Ward-Oriented Pharmacy", an on-ward service tailored to the Dutch hospital setting, will be developed. This service will consist of multifaceted interventions implemented in the Internal Medicine wards by hospital pharmacists. The effect of this service on preventable ADEs in elderly inpatients will be measured. Elderly patients are at high risk for ADEs due to multi-morbidity, concomitant disabilities and polypharmacy. Most studies on the incidence and preventability of ADEs in elderly patients have been conducted in the outpatient setting or on admission to a hospital, and fewer in the inpatient setting. Moreover, recognition of ADEs by the treating physicians is challenging in elderly patients because their disease presentation is often atypical and complex. Detailed information about the performance of the treating physicians in ADE recognition is scarce.

**Methods/Design:**

The design is a multi-centre, interrupted time series study. Patients of 65 years or older, consecutively admitted to Internal Medicine wards will be included. After a pre-measurement, a Ward-Oriented Pharmacy service will be introduced and the effect of this service will be assessed during a post-measurement. The primary outcome measures are the ADE prevalence on admission and ADE incidence during hospital stay. These outcomes will be assessed using structured retrospective chart review by an independent expert panel. This assessment will include determination of causality, severity and preventability of ADEs. In addition, the extent to which ADEs are recognised and managed by the treating physicians will be considered.

**Discussion:**

The primary goal of the WINGS study is to assess whether a significant reduction in preventable ADEs in elderly inpatients can be achieved by a Ward-Oriented Pharmacy service offered. A comprehensive ADE detection method will be used based on expert opinion and retrospective, trigger-tool enhanced, chart review.

**Trial registration:**

ISRCTN: ISRCTN64974377

## Background

Harmful events caused by medication are a widely recognised problem, both in hospital and outpatient settings [1-5]. These events are known as Adverse Drug Events (ADEs), usually defined as 'any injury due to the use of medication'. ADEs may occur during the normal use of medication as a result of an unavoidable pharmacological effect (side effects or Adverse Drug Reactions (ADRs)), or as a result of a medication error (preventable ADEs) [6]. Preventable ADEs are associated with substantial morbidity, increased mortality, a longer length of stay in the hospital and costs [7-9].

To increase medication safety, many interventions to reduce preventable ADEs have been studied and were found to be successful [6,10,11]. Full-time on-ward participation of clinical pharmacists in medical teams is an effective intervention in the hospital setting [12-15]. Nowadays, these services, often referred to as "Clinical Pharmacy", are routinely offered in most Anglo-Saxon countries [16-18]. In The Netherlands however, the Hospital Pharmacy organisation is mainly product-oriented. Also, most of the hospital pharmacists' time is taken up by activities such as quality assurance of medication compounding, verification of parenteral medication prepared by pharmacy technicians, therapeutic drug monitoring and medication logistics. In addition, the number of Dutch hospital pharmacists is low. On average, there are fewer hospital pharmacists per 100 hospital beds (0.75) in comparison to United Kingdome (1.42) and United States of America (14.1) [19]. As a consequence, routine clinical activities by Dutch hospital pharmacists are usually limited to off-ward services such as an on-call duty for consultations and checking of automated alerts pertaining to drug-drug interactions, drug-drug duplications and overdosages. The disadvantage of such a back-office organisation is that it gives hospital pharmacists limited insight into the relevant medication risks incurred by patients in the wards. The substantial incidence of preventable ADEs in Dutch hospitals found in recent studies [20-23] warrants an extension of the current off-ward clinical activities by an on-ward pharmacy service. To maximise its effectiveness, such a service should primarily be aimed at the highest medication risks both at patient and organisation levels. For efficiency reasons, an adaptation of Clinical Pharmacy to the Dutch hospital setting is needed because Dutch hospital pharmacists are scarce.

In this study, we choose to limit the on-ward pharmacy service to elderly hospitalised patients using five or more medications on the day of admission. Especially in elderly patients, pharmacotherapy management is challenging as polypharmacy, multi-morbidity and concomitant disabilities are often present. Altered physiological functions and cognitive decline even further increase the risk for ADEs in this vulnerable patient group [24-26]. A number of studies have described ADE incidence and preventability in elderly patients in the outpatient setting and on admission to hospitals [27-33]. However, limited data exist regarding these outcomes in the inpatient setting [34-36]. Furthermore, none of these studies have addressed the extent to which ADEs detected by the researchers were also recognised and managed by the treating physicians in the wards. Correct diagnoses in elderly patients are difficult to make because such patients often present with atypical symptoms [37]. One study found that only 51% of ADEs were recognised by the treating physicians [38]. However, the generalisability of these results to the inpatient setting is limited, because only Emergency Department physicians were involved. It thus appears that our knowledge on ADE recognition and management in the elderly inpatients is limited and further study is needed.

Numerous studies have investigated the effect of on-ward pharmacy services in the elderly hospital population by measuring surrogate end-points, such as inappropriate prescribing [39-42]. Only a few studies have measured clinical outcomes such as the number of ADEs [43,44]. However, the measurements conducted in these studies were limited to one specific type of preventable ADEs (e.g only coagulation-related) [44] or limited in the number of participants and conducted in one academic hospital [43]. Thus, the results of these studies cannot readily be generalised to other hospital settings.

The WINGS study is a multicentre study, designed to assess the reduction of ADE incidence in a high-risk population deploying limited pharmacist resources, focussing on ADE recognition and management by treating physicians, using clinical outcome as an effect measure. The on-ward pharmacy service designed in this study will be called "Ward-Oriented Pharmacy" as opposed to Clinical Pharmacy because hospital pharmacists will be attributed only part-time to this service.

The primary goals of this study are 1) to determine the number of ADEs on admission and during hospital stay in elderly patients, and to assess their causality, preventability, severity, recognition and management of ADEs by treating physicians, 2) to design and implement an effective and feasible on-ward pharmacy service for elderly patients in the Dutch hospital setting and 3) to measure the effect of this service on the incidence of preventable ADEs.

## Methods/Design

### Study design

The design is a multi-centre interrupted time series study. The interrupted time series (ITS) model follows the Cochrane Effective Practice and Organisation of Care Review Group criteria for short time series [45]. This type of series consists of pre- and post-intervention phases and needs to have at least three observation points in the pre-intervention phase and three in the post-intervention phase. Every data point needs to have at least 30 observations. The six data points needed will be strategically spread over pre- and post-measurement periods. The study will be conducted during a period of three years. An advantage of an ITS design is that it allows for the statistical investigation of potential biases such as secular trends in the estimate of the effect of the intervention [46]. ITS is also more feasible than a Randomized Controlled Trial in measuring the effect of interventions that require organisational change to a health care delivery system, which is the case in the WINGS study [45].

### Study setting

The participating hospitals are one academic hospital, the Academic Medical Centre in Amsterdam, and two non-academic hospitals, the Westfriesgasthuis Hospital in Hoorn and the Spaarne Hospital in Hoofddorp, the Netherlands. All internal medicine wards of these hospitals participate in the study.

### Study population

All consecutive patients of 65 years or older with an expected length of stay of 24 hours or longer, using 5 or more medications on the day of hospital admission and admitted to an internal medicine ward during measurement phases will be included. Patients admitted for scheduled chemotherapy, radiation therapy or transplantation, as well as patients transferred from another hospital or from a ward other than an internal medicine ward within the same hospital will be excluded. No patient will be included more than once.

### Intervention

During the whole study, all regular off-ward services, as described in the Introduction section, will be offered to Internal Medicine wards by the participating Hospital Pharmacy departments. During the intervention period, a designed Ward-Oriented Pharmacy service will be added to regular off-ward pharmacy services offered to all Internal Medicine wards of the participating hospitals. A Ward-Oriented Pharmacy service will consist of multifaceted interventions such as pharmacotherapy education, writing and implementation of drug protocols, face-to-face pharmacotherapy guidance of prescribers, participation in ward-rounds and medication reviews. Multifaceted interventions are reported to be more effective than single interventions, especially when these are complementary and not overly complex [47]. To promote implementation of a Ward-Oriented Pharmacy service, the three participating hospitals can choose different types of interventions to include in this service.

To be able to choose efficient interventions and to design a feasible Ward-Oriented Pharmacy service, the following steps will be taken:

1) An analysis of preventable ADEs based on the pre-measurement results will be conducted to identify most frequent medication risks in elderly inpatients. These risks will be categorised in patient-related medication risks and organisational medication risks. Patient-related medication risks refer to patients' characteristics, for example use of specific medication or the presence of specific co-morbidities, which might be associated with a higher risk for preventable ADEs. Organisational risks refer to ward characteristics, for example the extent to which pharmacotherapy guidelines or protocols are available and the level of existing knowledge and education. Lacking or insufficient pharmacotherapy guidelines, protocols or knowledge could entail a higher risk for preventable ADEs.

2) The results from step one will be presented to a multidisciplinary group consisting of Internal Medicine and Hospital Pharmacy staff members and residents of all three participating hospitals. These groups will prioritise risks and choose interventions tailored to local needs and possibilities to design a specific Ward-Oriented Pharmacy service. Tools and strategies like Bow-Tie risk analysis and the Swiss Cheese model [48,49] will be used to structure this process.

3) To further assure a successful implementation process, a finally designed Ward-Oriented Pharmacy service will be presented to all personnel in the participating departments. A one-month introduction period will be used to fine-tune the interventions. To keep the participating departments informed about the progress of the study, periodic evaluations will be planned and a newsletter will be distributed. After 7 months we expect the implementation of a Ward-Oriented Pharmacy service to be finalised.

### Study Outcomes

The primary outcomes of this study are 1) the number, severity and preventability of ADEs present at admission calculated per 100 hospitalisations, 2) the number, severity and preventability of ADEs during hospital stay calculated per 100 hospitalisations, 3) the percentage of ADEs recognised and appropriately managed by the treating physicians.

The secondary outcomes of this study are 1) the number of medication errors per number of medication orders and 2) the number of readmissions within three months after the index hospitalisation.

Depending on the type of the interventions that will be implemented, additional secondary outcomes will be measured to monitor the implementation of these specific interventions. For example, pharmacotherapy consultations given by hospital pharmacists on the wards can be monitored by recording the number of advices given to the physicians and nurses.

### Data collection and outcome assessment

**Figure 1:**
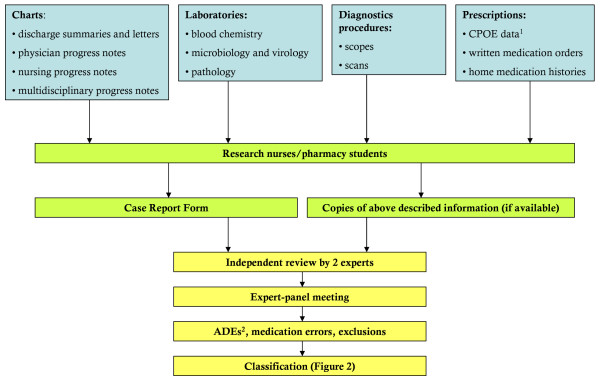
**Process of data collection and outcome assessment**. ^1 ^CPOE, Computer Physician Order Entry ^2 ^ADEs, Adverse Drug Events (preventable and non-preventable)

**Figure 2:**
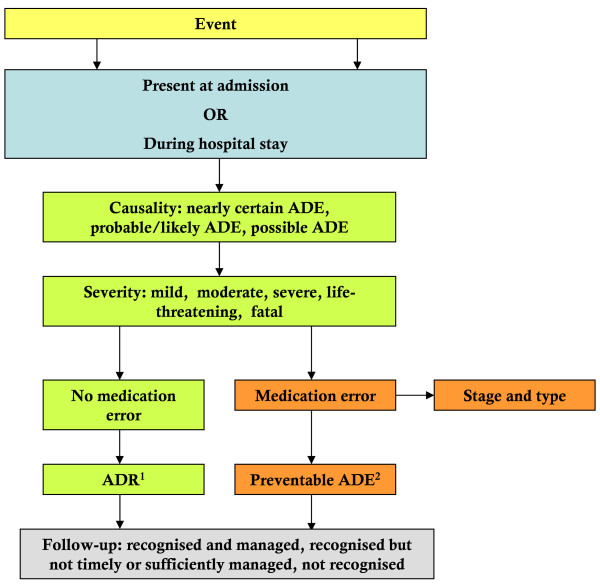
**Process of outcome classification and scoring**. ^1 ^ADR, Adverse Drug Reaction, non-preventable ^2 ^ADE, Adverse Drug Event (preventable and non-preventable)

The flow charts of the data collection process and the outcome assessment process are shown in Figures 1 and 2 and have been adapted from previous studies [50,51].

First, trained research nurses and pharmacy students will collect all information available about the index hospitalisation of included patients, after discharge of each patient. All charts, laboratory results, rapports of diagnostic procedures and medication prescriptions will be assembled. A Case Report Form (CRF) will be completed for every included patient. In the CRF, a selection of ADE triggers is listed and can be checked off when applicable. The selection of included ADE triggers was based on available trigger tools [52,53] and expert opinion.

Second, copies of the gathered information and the CRF of all included patients will be presented to two independent experts: a senior specialist in Internal Medicine and a senior clinical pharmacist specialised in geriatrics (LA and CS respectively). The two experts will first review the presented information independently from each other. This first review is an implicit process based on expert judgment, which is still the "gold standard" in adverse events determination [52]. In this study we use implicit judgment of a pharmacist and physician team because their knowledge has shown to be complementary [54,55].

Third, the two experts will discuss their findings in an expert panel meeting. During this second review, the causality between the adverse events found during the first review and commission or omission of medication will be assessed. Only ADEs for which the experts meet consensus on causality will be recorded and subsequently assessed on preventability, severity and, when applicable, on type of medication error. If consensus cannot be reached, the opinion of a third expert will be sought.

For the ADE causality assessment used in the second review, we developed a structured method based on the World Health Organization - Uppsala Medical Centre (WHO-UMC) system [56]. The causality will be scored on a 3-point scale: nearly certain (> 90% certainty that there is a causal relationship between adverse event and a drug), probable/likely (> 65-90% certainty that there is a causal relationship between adverse event and a drug), possible (33-65% certainty that there is a causal relationship between adverse event and a drug). ADEs with 32% or less certainty in causality will not be recorded and therefore not further assessed. The severity of ADEs will be scored according to the Common Terminology Criteria for Adverse Events version 3.0 (CTCAEv3) developed by the U.S. National Cancer Institute [57]. CTCAEv3 is a descriptive terminology used to report adverse events in many clinical trials. For each ADE, a 5-points scale of seriousness is included: mild, moderate, severe, life-treating, fatal.

An innovative item in our ADE assessment is the determination of recognition and management of ADEs by the treating physicians during hospitalisation. For every ADE present on admission or occurred during the index hospitalisation, the experts will assess if it was recognised by the treating physicians in the Internal Medicine wards. When applicable, the experts will assess whether the chosen management was timely and sufficient to stop or preclude further harm.

**Figure 3:**
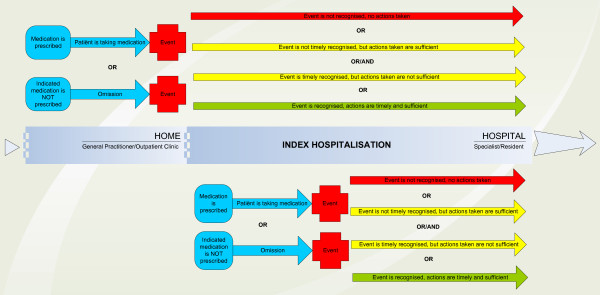
**ADEs included in the study**.

ADEs that will be included in this study are shown in Figure 3. The index hospitalisation is the hospitalisation sampled. ADEs will be included if they were present on admission or occurred during the index hospitalisation. For each included patient, one of the experts (CS) will also score a predefined set of medication errors that occurred during the index hospitalisation, but did not cause patient harm. Medication errors will be classified according to the Dutch Central Medication Incidents Registration.

### Definitions

The definitions used in this study were adapted from the Glossary of Terms Related to Patient and Medication Safety by Expert Group On Safe Medication Practices [6].

*Adverse Drug Event *(ADE) is any injury occurring during the patient's drug therapy and resulting either from appropriate care, or from unsuitable or suboptimal care. ADEs include non-preventable *Adverse Drug Reactions *(ADRs) during normal use of medication, and any harm secondary to a medication error (preventable ADEs), both errors of omission and commission will be included. An ADE can result in different clinical outcomes, for example: abnormal laboratory values, worsening of the existing disease, lack of any expected disease improvement, or outbreak of new symptoms or diseases.

*Harm*: temporary or permanent impairment of the physical, emotional, or psychological function or structure of the body and/or pain resulting there from requiring intervention. In this study also abnormal laboratory values will be counted as harm.

*Medication error*: any preventable event that may cause or lead to inappropriate medication use or patient harm while the medication is in the control of the health care professional or patient. Such events may be related to professional practice, health care products, procedures, and systems, including prescribing; order communication; product labelling, packaging, and nomenclature; compounding; dispensing; distribution; administration; education; monitoring; and use.

### Sample size calculation and data analysis

The power-calculation for this study was based on the expected incidence of 15 ADEs per 100 hospitalisations and a clinically relevant 50% reduction by a Ward-Oriented Pharmacy service in the post-measurement period. This assumption was made based on the findings of two controlled studies that assessed active clinical pharmacists participation in medical teams on the wards, showing a reduction of 66% and 78% respectively [12,13]. To be able to identify a significant reduction from 15 ADEs to 7.5 ADEs per 100 hospitalisations, 496 patient admissions are needed, equally divided between pre- and post-measurement period (α = 0,05 and β = 0,8). This sample size enables to identify a reduction of 19 ADEs or more from the expected 38 ADEs in 248 admissions during the pre-measurement period.

Descriptive statistics will be used to summarise patient characteristics for the three participating hospitals and to check for any imbalance in variables between pre- and post-measurement groups. By comparing the pre- and post-measurement periods, the effect of a Ward-Oriented Pharmacy service will be analysed using a suitable generalised linear model for a short interrupted time series. Adjustment will be made for any case mix imbalances between pre- and post-measurement periods and background trend in the ADE rate over time.

Exploratory analyses are also planned to examine the effect of factors like age, sex, length of hospital stay, Charlson Co-morbidity Index Score [58], renal function on the primary and secondary outcomes. For this purpose unadjusted univariate and adjusted multivariate Poisson regression analyses are planned.

### Quality assurance

To ensure quality of data collection, research nurses and pharmacy students will be trained by an experienced member of the research team (JK) to collect the data and how to complete the CRFs. Before the start of the study, research nurses and pharmacy students involved in data-collection will test whether the designed CRF is explicit, comprehensive, and user-friendly. When necessary, the CRF will be adjusted to improve it and a manual will be written to guide the data-collectors during the whole process. This manual will also be tested to assure that it is explicit, comprehensive, and user-friendly.

Before the start of the study, the structured outcome assessment by the expert panel will be tested by the experts on a sample of 10 patients to ensure that it is explicit, comprehensive and user-friendly. If necessary, the assessment will be adjusted to improve these characteristics. Furthermore, the intra-rater and inter-rater reliability of the assessment process by the expert team will be assessed. For this purpose the kappa statistic (κ) will be calculated. A kappa value of 0.00 will be considered as poor agreement, 0.01-0.20 as slight agreement, 0.21-0.40 as fair agreement, 0.41- 0.60 as moderate agreement, 0.61-0.80 as substantial agreement, and 0.81-1.00 as almost perfect agreement [59].

### Study organisation and management

The research protocol was submitted to the Medical Ethics Committee of the Academic Medical Centre (AMC) before the start of the study. The Medical Ethics Committee of AMC judged the protocol as not needing an approval because the Dutch Medical Research Involving Human Subjects Act (WMO) does not apply to the WINGS study. In this research, we use a retrospective chart review to evaluate the effect of an intervention aimed at quality improvement. Therefore, the integrity of the patients is not influenced. All patient data will be analysed anonymously by coding every patient by a 6-digit number.

In every hospital a group of representatives of the Internal Medicine Department and Hospital Pharmacy Department has been formed. The main investigator (JK) and project manager (LL) will meet at least monthly with these representatives to oversee the progress of the study.

The WINGS study is part of the CAREFUL (pharmacist Coordinated ADE Reducing Efforts For Use in all Levels of healthcare) research programme, based on a cooperation of Leiden University Medical Centre, Academic Medical Centre Amsterdam, University Medical Centre Groningen and University Medical Centre Utrecht/Utrecht University. Within this programme, interventions are studied that are aimed at promoting medication safety in hospital and outpatient settings.

## Discussion

Clinical Pharmacy as practised in Anglo-Saxon countries has shown to be successful in reducing preventable ADEs in various hospital patient populations [12-15]. In this study, the effect of a Ward-Oriented Pharmacy service on preventable ADEs in elderly inpatients will be investigated. The Ward-Oriented Pharmacy service is an on-ward pharmacy service based on the successful Clinical Pharmacy practice but tailored to the Dutch hospital setting. The Ward-Oriented Pharmacy service will include multifaceted interventions implemented in Internal Medicine wards by hospital pharmacists. Adapting Clinical Pharmacy practice to the Dutch hospital setting is necessary, because Clinical Pharmacy entails a full-time participation of clinical pharmacists in medical teams on the wards, a time investment that is not feasible within the current organisation of Hospital Pharmacy in the Netherlands. This applies also to many other European countries [19]. Moreover, active on-ward participation of pharmacists in medical teams is a practice Dutch physicians are not familiar with. This could raise barriers that also need to be considered.

The results of the WINGS study will add insight into the effectiveness of Ward-Oriented Pharmacy on the reduction of ADEs in elderly inpatients. Also the extent to which this service is feasible in the Dutch setting will be explored. By measuring ADEs across the process of admission and subsequent hospitalisation, the extent to which ADEs are recognised and appropriately managed by treating physicians will be investigated. Especially in elderly patients, ADE awareness is essential to be able to practice the safest possible pharmacotherapy [37].

The method of data collection and chart review used in the WINGS study differs from the standard trigger-tool based chart review method used in other ADE studies [35]. According to the standard method, only charts with one or more triggers are subjected to further review by a physician. Therefore, only a selection of ADEs can be scored and this selection depends on the predefined selection of triggers used in the first step of data collection and examination procedure. In contrast, the trigger-tool based CRF in this study serves only as an aid to help the experts. Trigger-tools have shown to be able to increase the detection of ADEs and can be computerised or used manually [60]. We will use manual screening for ADE triggers in patient files instead of electronic trigger-tools, because by the manual method, the narrative information, such as progress notes or discharge letters, can be screened. Symptoms like constipation, dizziness, falls and hypotension are examples of ADE triggers in narrative information sources. By subjecting all included patients to further review and applying the above described method, a more sensitive ADE assessment by the experts is expected. In our setting, where the magnitude of medication risks in elderly inpatients is unclear, such a comprehensive approach is needed to gain detailed insight into this problem. The time-consuming character of our method is not a barrier, because it is used for research purposes.

In the WINGS study, the primary outcome (ADE) is assessed by using a combination of implicit expert judgment with structured causality assessment based on the WHO-UMC system [56]. Because the WHO-UMC system was developed for ADRs causality assessment, we added or discarded items to design a causality assessment strategy for ADEs and therefore assessment of ADRs and preventable ADEs. Furthermore, both errors of omission and commission are considered as causes of preventable ADEs in our assessment [50].

The expert judgment is the most popular and most widely used method, even given limitations like lack of reproducibility, poor inter- and intra-rater agreement and a lack of standardised clinical evaluation. Algorithms for ADR causality assessment have been developed to overcome the limitations of expert judgment [61]. However, as mentioned before, ADE assessment in elderly patients could be complicated by factors like multi-morbidity, polypharmacy and atypical presentation of diseases. Therefore, opinion of clinically experienced reviewers, who are able to weigh drug causation considering all these factors, is essential. In order to do so, less flexible and less specific algorithms are not suitable in this study. It has also been shown that reproducibility of results from the use of such algorithms can drastically decrease, yielding low inter-rater variability, because clinical judgment is always necessary to be able to answer all the questions included [61].

To improve the validity of the method used in this study, we have taken three measures:

1) by using a CRF we help the experts to standardise their clinical evaluation, 2) by combining implicit review followed by structured causality assessment we lower the subjectivity of the expert's decisions 3) by deployment of the same expert team in ADE assessment in the pre- and post-measurement phases the problem of low inter-rater agreement seen in studies using expert judgment can be partly overcome [55].

## Competing interests

The authors declare that they have no competing interests.

## Authors' contributions

JK, PW, SM, SdR and LL conceptualised the study and developed the study design and methods. JK, PW and SM drafted the protocol. JK is responsible for data collection and overseeing the running of the study. LA, CS and PK helped in developing of methods for data-collection and outcome assessment used in this study. LA and CS will access the outcomes of the study. MD helped with an analysis-plan and drafting of the protocol.

All authors read and approved the final manuscript.

## Collaborators

Joost L.B. Hoekstra, MD, PhD, Department of Internal Medicine and Minke E.P. Jansen, PharmD, MSc, Department of Hospital Pharmacy, *Academic Medical Centre, Amsterdam, The Netherlands*. Wim G. Meijer, MD, PhD, Department of Internal Medicine and Bea M. van der Kleij, PharmD, MSc, Department of Hospital Pharmacy, *Westfriesgasthuis Hospital, Hoorn, The Netherlands*. Anne M. Lagaay, MD, PhD, Department of Internal Medicine and Geriatrics, *Spaarne Hospital, Hoofddorp, The Netherlands *and Ruud T.M. van der Hoeven, PharmD, MSc, Director of Pharmacy Foundation of Haarlem Hospitals, *Haarlem, The Netherlands*.

## Pre-publication history

The pre-publication history for this paper can be accessed here:

http://www.biomedcentral.com/1472-6963/11/124/prepub
